# Existentialism and My ‘Postwolf’ Dachshund: Authenticity in the Age of Genetic Engineering

**DOI:** 10.1111/bioe.13428

**Published:** 2025-06-25

**Authors:** Donrich Thaldar

**Affiliations:** ^1^ School of Law University of KwaZulu‐Natal Durban South Africa

**Keywords:** authenticity, CRISPR, existentialism, genetic engineering, identity, posthumanism

## Abstract

Human genetic engineering has the potential to profoundly alter the traits of future generations, raising critical ethical questions about authenticity and identity. Essentialist perspectives reject genetic engineering, claiming it inherently compromises authenticity by deviating from a species‐typical genome. In contrast, this article advocates for an existentialist interpretation of authenticity, drawing on the philosophies of Heidegger and Sartre. Here, authenticity is understood as a dynamic and relational process rooted in individual choice, responsibility, and engagement with existential conditions. Unlike essentialism, existentialism evaluates genetic interventions not as inherently wrong but based on their alignment with values such as autonomy and authenticity, offering a more flexible and ethically robust framework. Existentialism's emphasis on individual freedom, self‐determination, and the creation of meaning in life makes it ethically more compelling than essentialist frameworks, which impose deterministic constraints. Moreover, essentialist critiques falter when they concede the permissibility of therapeutic genetic engineering, undermining the notion of an inherently valuable species‐typical human genome. In contrast, existentialism affirms the transformative potential of genetic engineering, recognising it as a means to expand autonomy, self‐expression, and opportunities for flourishing when applied responsibly. The article advocates for a balanced ethical approach by integrating the Principle of Procreative Beneficence, which promotes enhancements to optimise flourishing, with the Principle of Procreative Non‐Maleficence, which safeguards autonomy by preventing deterministic constraints. This complementary framework, grounded in an existentialist perspective, reframes authenticity as an evolving concept aligned with the transformative possibilities of genetic engineering, enriching the discourse on bioethics and identity in a rapidly changing era.

## 1 Introduction

What does it mean to be authentic in an age where genetic engineering can alter the fundamental aspects of human existence? Consider my dachshund, Zazu—a far cry from his wolf ancestors, with his short legs, elongated body, and a temperament suited to companionship rather than predation. His traits, shaped by generations of selective breeding, might prompt the question: has he lost his ‘authentic wolfness’? Similarly, the rapid advancements in genetic engineering compel us to ask the following: do transformations wrought by biotechnologies undermine authenticity, or do they simply reflect the dynamic nature of existence?

Advancements in genetic engineering, driven by ground‐breaking technologies like CRISPR‐Cas9 and the more refined prime editing, have rapidly accelerated, enabling precise genome alterations with the potential to cure genetic disorders and opening the door to human enhancement. While these innovations promise profound benefits, they also raise critical bioethical questions that challenge not only our physical boundaries but also our philosophical and ethical understandings of identity. Authenticity has emerged as a pivotal concept in contemporary bioethics, as genetic interventions extend beyond altering physical traits to potentially influencing personality, cognition, and behaviour, thereby impacting an individual's capacity for self‐determination and authentic living. The prospect of designing or enhancing specific traits highlights the ethical tension between technological possibilities and the risk of undermining individual authenticity.

This raises a critical research question: Can genetic engineering be reconciled with authenticity? Addressing this question is crucial for shaping ethical practices and policies in the application of genetic technologies. To explore this issue, the article examines the philosophical foundations of authenticity through the perspectives of classical existential philosophers Martin Heidegger and Jean‐Paul Sartre. By integrating their insights with the ethical challenges posed by genetic engineering, it aims to enrich the bioethical discourse and guide responsible biotechnological practices.

## 2 Existentialism: A Framework for Authenticity in Genetic Engineering

Genetic engineering poses unique challenges to our understanding of authenticity, particularly when considered through the lens of essentialist and existentialist philosophies. Essentialism roots authenticity in adherence to an unaltered human essence, often defined by fixed biological or genetic characteristics. From this perspective, deviations from species‐typical traits through genetic engineering are seen as threats to the moral and existential integrity of individuals. Such a view suggests that altering this essence undermines both identity and dignity.

In contrast, existentialist thought offers a dynamic and relational view of authenticity, emphasising the ongoing process of engaging with life's conditions to shape one's existence in alignment with self‐determined values. The existentialist framework articulated by Martin Heidegger and Jean‐Paul Sartre provides insights into how genetic engineering can expand, rather than diminish, the capacity for authentic living by fostering autonomy, personal responsibility, and the creative transformation of life's givens.

This section explores the existentialist view of authenticity through specific concepts and their application to genetic engineering. By integrating Heidegger's emphasis on thrownness and Sartre's notion of radical freedom, it critically examines how genetic interventions intersect with autonomy and authenticity. Additionally, the relationship between these values is further clarified through contemporary narrative accounts, offering a robust philosophical foundation for evaluating the ethical dimensions of genetic engineering.

### 2.1 Heidegger

Heidegger's concept of *authenticity* (*Eigentlichkeit*), articulated in his groundbreaking work *Being and Time*, is rooted in the idea of taking ownership of one's existence by confronting life's finite nature and striving to live in accordance with one's ownmost potential [1]. At the heart of this framework is the concept of *thrownness* (*Geworfenheit*)—the recognition that humans are always born into specific conditions that shape, but do not determine, their potential. These conditions, including genetic traits, are not barriers to authenticity but part of the foundational *givens* individuals must meaningfully engage with to define themselves. This perspective underscores that authenticity is not about escaping one's conditions but about engaging with them critically and meaningfully, integrating them into one's possibilities for being.

For Heidegger, authenticity is not about rejecting these formative influences or striving to escape them. Instead, it arises by critically engaging with and reinterpreting these influences, integrating them into one's possibilities for being. Critics of genetic engineering often contend that such interventions compromise a child's agency. Heidegger's framework challenges this view by underscoring that agency and authenticity emerge from actively taking ownership of one's existence, making choices that shape identity in dialogue with the conditions one inherits, rather than being defined by them.

A key element of Heidegger's philosophy is the *call of conscience*, which emphasises the relational and dynamic nature of authenticity. The call of conscience confronts individuals with the gap between their current state and their potential for self‐realisation, urging them to take responsibility for their choices and embrace their possibilities for being. In the context of genetic engineering, this concept resonates profoundly, highlighting the ethical and existential responsibilities borne by both (a) the parents who employ genetic engineering and (b) the resulting genetically engineered offspring. Parents, in making genetic decisions, must consider whether their choices enable their child's capacity for authentic living. This involves creating conditions that do not predetermine a child's path but rather support their future autonomy and capacity for self‐expression. As children grow, they too bear responsibility for engaging with the conditions shaped by these decisions, integrating them into their evolving narrative of selfhood.

Authenticity, therefore, is not an individual pursuit but a shared responsibility, reflecting the intergenerational dimensions of existentialist ethics. It is not merely about passing down genetic traits but about fostering an environment where the child's potential for authentic self‐realisation can flourish. Through this lens, genetic engineering becomes not an act of control, but one of commitment to shared existential possibilities.

### 2.2 Sartre

Sartre's existentialist philosophy, as developed in *Being and Nothingness*, introduces a critical tension between *facticity*—the unchangeable givens of one's existence—and *transcendence*—the freedom to surpass these givens through conscious action [2]. For Sartre, authenticity arises when individuals embrace this tension, recognising that while facticity constrains them, it does not determine their essence. Authenticity, in Sartre's view, is not about escaping facticity but about transforming it into a meaningful foundation for self‐creation. Sartre's emphasis on freedom does not negate the influence of facticity but rather positions it as a necessary condition for authentic self‐creation, especially in contexts of transformation and ethical decision‐making.

Central to Sartre's philosophy is the concept of *radical freedom*, the idea that individuals are always free to define their essence through conscious choices, regardless of the constraints imposed by their circumstances. Sartre emphasises that this freedom is not without weight—it entails an equally radical responsibility to take ownership of one's choices and their consequences. As Sartre explains in *Existentialism is a Humanism*, freedom is accompanied by profound responsibility, both for oneself and for the broader consequences of one's choices [3]. This notion of radical freedom confronts the existential anxiety that arises from the awareness of being solely responsible for shaping one's identity.

In the context of genetic engineering, Sartre's concept of radical freedom offers a profound counterpoint to essentialist arguments that view genetic interventions as inherently constraining autonomy and precluding authenticity. Sartre's perspective reveals that genetically engineered traits, as elements of facticity, do not determine the essence of individuals but instead form part of the conditions they navigate and transcend through their radical freedom. This ongoing project of becoming enables individuals to critically engage with their genetic givens, reinterpreting and transcending constraints in alignment with their self‐determined aspirations. By framing authenticity as a dynamic and evolving endeavour, Sartre's philosophy challenges essentialist critiques, highlighting that genetic engineering, far from being inherently limiting, can foster authentic identity formation when approached thoughtfully.

Sartre's concept of *bad faith* further illuminates the dynamic between freedom and the responsibilities associated with procreative decisions. Bad faith arises when individuals deny their freedom or responsibility, attributing their actions to external forces. In the context of genetic engineering, once safe, effective, and accessible technologies become available, parents cannot ignore their freedom to thoughtfully engage with the possibilities these advancements offer. Like plucking an apple from the scientific tree of knowledge, the availability of genetic engineering represents a milestone that cannot be reversed. Once this metaphorical apple has been plucked, parents must confront the reality of its existence and decide whether to use it and, if so, to effect what kind of genetic changes. These decisions must be made thoughtfully, always considering how shaping children's facticity—the givens of their lives—will impact their autonomy and capacity for authentic self‐realisation. Sartre's framework challenges parents to critically and responsibly engage with these technologies, recognising that avoidance or denial of their transformative potential is itself a choice, and one that risks falling into bad faith. This call for critical engagement reflects Sartre's broader argument in *Existentialism is a Humanism* that individuals cannot evade their freedom without ethical consequence [3].

In conclusion, Sartre's existentialist philosophy provides a nuanced framework for understanding authenticity in the age of genetic engineering. By emphasising the interplay between facticity and transcendence, Sartre highlights the potential for individuals to navigate and transform the givens of their existence, including those shaped by genetic interventions, into opportunities for authentic self‐realisation. His concept of radical freedom underscores the ethical weight of parental responsibility in procreative decisions, challenging parents to thoughtfully engage with the transformative possibilities offered by genetic engineering rather than retreating into bad faith. Far from being inherently limiting, genetic engineering, when approached with a commitment to fostering autonomy and authenticity, can enrich the conditions for human flourishing. Sartre's insights remind us that authenticity is not about preserving an unchanging essence but about creatively transcending constraints to shape a meaningful and self‐determined existence.

### 2.3 Authenticity and Autonomy

Building on the existentialist perspectives of Heidegger and Sartre, this subsection extends the discussion by focusing on the relationship between authenticity and autonomy—key values in evaluating the ethical dimensions of genetic engineering. Traditional ethical literature, such as Taylor's *The Ethics of Authenticity* [4], often presents authenticity as a condition for autonomy, suggesting that living authentically—by aligning one's actions and choices with one's ‘true self’—is necessary for making genuinely autonomous decisions. However, existentialist accounts, such as those of Iftode and colleagues,^1^ challenge this hierarchical framing. They argue that authenticity and autonomy are irreducible yet interdependent values, neither of which can be fully understood in isolation. This relational approach emphasises that the capacity to live authentically arises through autonomous choices, just as autonomy is enriched by living authentically.

Narrative accounts of autonomy, such as those articulated by Leuenberger [6], complement this perspective and provide the framework that this article adopts. Leuenberger's account frames autonomy as a dynamic process grounded in an individual's evolving life story. Rather than being defined by a static alignment with a ‘true self,’ autonomy involves the capacity to weave one's experiences, choices, and values into a coherent and meaningful narrative. This dynamic and relational understanding resonates with existentialist critiques of essentialism, highlighting that autonomy and authenticity are processes of engagement with one's facticity, shaped by both individual freedom and relational contexts.^2^ Together, these perspectives reject static, hierarchical framings and embrace the interplay between authenticity and autonomy as central to human flourishing in the context of genetic interventions [8].

The integrated view presented here—authenticity and autonomy as interdependent and mutually reinforcing—provides a nuanced ethical framework for understanding genetic interventions. Genetic engineering is reframed not as a risk to these values but as an opportunity to enrich them when approached responsibly. By aligning parental decisions with the principles of fostering self‐authorship and autonomy, this framework challenges deterministic fears while emphasising the ethical responsibility to safeguard the child's capacity for meaningful choice and self‐definition. This perspective deepens the existentialist critique of essentialism, positioning genetic engineering as a potential tool for fostering human flourishing in a way that honours both authenticity and autonomy.

### 2.4 Ethical Weight and Existential Lightness

Existentialism offers a cohesive framework for understanding authenticity in the context of genetic engineering, integrating the philosophies of Heidegger and Sartre. Heidegger highlights thrownness and the call of conscience, and Sartre frames authenticity as an ongoing process of transcending constraints through conscious action. Together, these perspectives present genetic engineering not as a threat to authenticity but as an opportunity for authentic self‐realisation when approached responsibly. Parental genetic decisions shape the givens into which children are born, but these givens are neither deterministic nor limiting. Authenticity emerges as both parents and children critically engage with these conditions, transforming them into opportunities for growth and self‐definition.

Unlike essentialism, which categorically rejects genetic engineering as inherently wrong, existentialism evaluates such interventions based on their alignment with autonomy and authenticity. While genetic engineering can foster opportunities for flourishing and self‐definition, existentialist ethics cautions against interventions that impose deterministic constraints or fail to respect a child's capacity to engage meaningfully with their givens. The existentialist approach, therefore, does not judge genetic engineering as inherently wrong or right but calls for a reflective evaluation of its broader ethical implications.

Based on the analysis of Heidegger and Sartre above, this article defines *authenticity* as the alignment between one's actions, desires, and life choices with an evolving identity, while *identity* is understood as the traits, characteristics, and experiences that shape an individual's sense of self.^3^ Within this existentialist framework, genetic interventions are not deterministic constraints but part of the givens into which a child is born. Authenticity emerges as parents and children critically and reflectively engage with these conditions, transforming them into opportunities for growth and self‐definition.

Importantly, from the analysis in this section, a fundamental reason why existentialism is ethically more attractive than essentialism crystallises: it prioritises individual freedom, self‐determination, and the dynamic process of creating meaning in life. Sartre's assertion that ‘existence precedes essence’ encapsulates this perspective, emphasising that individuals are not bound by predetermined purposes but must actively shape their own identities [3]. Essentialism, in contrast, risks deterministic implications by suggesting that individuals are defined, entirely or significantly, by their biology or heritage. By rejecting such determinism, existentialism recognises the potential for individuals to transcend their facticity—the given circumstances of their existence—through freedom, choice, and self‐reflection.

To better illustrate the contrast between the essentialist and existentialist perspectives on authenticity and related concepts, I present a summary table below. This table highlights the key differences in how each view conceptualises authenticity, human nature, genetic engineering, and identity, offering a clearer lens through which to evaluate the ethical implications of genetic interventions (Table 1).

**Table 1:** Comparison of essentialist and existentialist views.

Aspect	Essentialist view	Existentialist view
Authenticity	Authenticity is tied to a fixed, unchanging human essence, often rooted in biology.	Authenticity is a dynamic, relational process of self‐realisation, shaped by choices.
Human nature	Human nature is pre‐determined and should remain unchanged.	Human nature is fluid and evolves through individual choices and external influences.
Genetic engineering	Genetic modifications undermine authenticity by disrupting the ‘true’ essence of humanity.	Genetic engineering can support authenticity by expanding opportunities for autonomy and flourishing.
Identity	Identity is rooted in biological or genetic traits, which should be preserved.	Identity is shaped by both genetic and environmental factors, with a focus on personal agency.
Autonomy	Constrained by genetic engineering, as it predetermines engineered individuals’ choices.	Impacted by how genetic engineering is applied—it may constrain, preserve, or enrich autonomy.
Ethical considerations	Genetic engineering is ethically problematic because it alters the human essence.	Ethical considerations focus on how interventions enable or constrain the ability to live authentically.
Philosophical foundation	A fixed conception of humanity.	Personal responsibility and freedom.

## 3 Challenging Essentialism

This section critically examines essentialist critiques of genetic engineering through the perspectives of Jürgen Habermas [11] and Francis Fukuyama [12]. It is important to note that essentialism encompasses a range of interpretations and is rarely adopted in its purest or most rigid form. While this article defines essentialism in its pure form—characterising genetic engineering as categorically wrong—it does not suggest that all essentialist thinkers fully adhere to this strict view. Indeed, Habermas and Fukuyama are not pure essentialists; both allow for therapeutic genetic engineering, which deviates from the strict essentialist stance. However, at the core of their arguments, their approaches remain fundamentally essentialist in nature, emphasising the preservation of a perceived human essence. This section begins by outlining their respective arguments and identifying foundational inconsistencies before presenting an existentialist critique that challenges their assumptions and offers an alternative framework for understanding the ethical implications of genetic interventions.

### 3.1 Habermas

Habermas critiques genetic engineering through a Kantian lens, arguing that parents acting as genetic designers impose an alien determination of identity on their child. He claims genetic interventions are ‘irrevocable’ and, unlike environmental influences such as education, infringe on the child's autonomy and dignity. While he permits therapeutic interventions by presuming the child's consent, he opposes non‐therapeutic enhancements, raising the critique: why assume a child would consent only to therapeutic interventions and not to non‐therapeutic enhancements that might enhance flourishing or autonomy? This inconsistency weakens his position. Furthermore, it is unclear why consent or assumed consent is ethically relevant or required when parents make decisions that will shape their prospective children's facticity. Parents are typically trusted to make all significant decisions on their young children's behalf—sometimes very much against the child's wishes—without the need to consider or construct some form of assumed consent. For example, decisions about medical treatments, schooling, or religious upbringing are routinely made by parents, even when these choices profoundly influence the child's life trajectory. By holding genetic interventions to a higher standard of ethical scrutiny, Habermas's argument appears inconsistent with the broader framework of parental responsibility and decision‐making.

Central to Habermas's argument is the notion that genetic factors form a person's ‘inner nature,’ reflecting an essentialist view that ties identity to an unchanging human essence. While he does not explicitly address authenticity, this perspective risks reducing identity to a biologically determined framework.^4^

Habermas's critique revolves around the notion of ‘irrevocability,’ which operates implicitly in both temporal and qualitative contexts. He contrasts genetic determinations with environmental influences, suggesting that the former are irrevocable over time. However, this distinction is unconvincing: both genetic traits and environmental factors, such as education or socialisation, are irrevocable once they occur. Individuals continuously engage with and reinterpret these givens, shaping their identities dynamically. Habermas also implies that genetic traits are qualitatively distinct, referring to them as a person's ‘inner nature’ and suggesting they hold a greater significance for identity than environmental factors. Yet, identity emerges through the interaction of genetic and environmental influences, neither of which can be deemed inherently more essential. This unexamined reliance on the idea of irrevocability weakens his critique, revealing its reliance on oversimplified assumptions.

Habermas's reliance on an essentialist conception of ‘inner nature’ overstates the determinative power of genetics and fails to acknowledge the dynamic, relational processes through which individuals navigate their givens to shape their identities. Consequently, his critique of genetic engineering becomes philosophically and logically untenable.

### 3.2 Fukuyama

Fukuyama critiques genetic engineering through the concept of an essential human quality, ‘Factor X,’ which he claims forms the basis of human dignity. Any disruption to Factor X—such as through genetic engineering—threatens this foundation, aligning Fukuyama's critique with essentialist assumptions that tie authenticity to an unchanging human essence.

Drawing on the philosophical tradition that links dignity to a perceived essential human nature, Fukuyama expands Kant's focus on rationality and free will to a holistic Factor X, describing it as the integration of multiple qualities, such as reason, emotions, sociability, and consciousness, that collectively define humanity. However, his failure to provide a definitive list of these qualities renders Factor X expansive yet ambiguous, leaving its boundaries open to interpretation.

Fukuyama's argument takes a controversial turn by romanticising human frailty and suffering. He suggests that the vulnerability associated with disease and limitations enriches human experience and adds meaning to life. If these aspects are integral to Factor X, then genetic interventions aimed at reducing suffering—such as eliminating cancer risk—might, under his framework, compromise dignity. This reasoning implies that preserving dignity requires maintaining human vulnerabilities, an inhumane conclusion that seems incompatible with dignity itself. Despite this, Fukuyama permits therapeutic genetic engineering but offers no explanation for why these interventions would not also compromise Factor X.

Fukuyama's essentialist framework also distinguishes between essential and non‐essential traits, classifying characteristics like natural talents, skin colour, and gender as ‘accidents of birth’ that do not influence dignity. Yet, his blanket prohibition of all non‐therapeutic genetic engineering is a significant non sequitur: if altering non‐essential traits does not affect Factor X, how can it undermine dignity?

In summary, while Fukuyama's Factor X seeks to provide a unified framework for human dignity, its ambiguities and contradictions weaken its ethical applicability to genetic engineering. By tying dignity to an unchanging human essence, his argument romanticises suffering while failing to justify the selective permissibility of therapeutic interventions.^5^

### 3.3 Existential Critique

From an existentialist perspective, the central flaw in the perspectives of Habermas and Fukuyama is their assumption that all (non‐therapeutic) genetic engineering is necessarily ethically problematic. This perspective overemphasises the determinative power of genetic interventions, overlooking the complex and dynamic interplay of factors that shape identity. Genetic engineering does not inherently fix or predefine a child's identity; rather, it introduces conditions into the child's *thrownness*—the givens of existence that must be navigated, interpreted, and redefined. The existential challenge lies not in avoiding genetic engineering altogether but in ensuring that such interventions align with an ethical framework that respects the child's autonomy, supports their capacity for self‐definition, and fosters their ability to live authentically.^6^

To illustrate, my dachshund, Zazu, whose ancestors were wolves, exemplifies how genetic transformation need not undermine authenticity. Generations of selective breeding have introduced traits far removed from his wolf heritage—short legs, an elongated body, and a temperament suited for companionship rather than hunting. Critics might argue that this transformation stripped Zazu of his ‘authentic wolfness’. However, his authenticity does not depend on preserving a ‘lupine essence’. Instead, it emerges from his active engagement with the conditions of his existence as a dachshund, shaped by deliberate human intervention. This highlights a fundamental existentialist insight: authenticity is not about adherence to an unchanging essence but about how one navigates and integrates the givens of life—whether shaped by nature or intentional transformation.

Similarly, human genetic engineering represents a continuation of humanity's broader adaptive processes, challenging the essentialist notion of a fixed human essence. Just as Zazu's transformation through selective breeding resulted in a new set of conditions he authentically engages with, genetic engineering introduces new givens into a person's existential landscape. These givens, whether natural or engineered, do not define a person's authenticity but rather set the stage for self‐determined engagement and transcendence. For instance, genetically enhanced children with improved cognitive or physical traits may navigate their lives with greater opportunities for flourishing, provided these enhancements do not impose deterministic constraints.

This perspective finds support in evolutionary biology, which demonstrates that species, including humans, are not static entities but are subject to continuous adaptation and change. As Proctor aptly notes, ‘If evolution has taught us anything, it is that there is no essence of humanity, no fixed and final form’. [16] Human genetic engineering, like evolution or selective breeding, introduces changes that expand rather than diminish the conditions within which authenticity is realised. The key ethical question, then, is not whether such transformations are inherently inauthentic but whether they enable individuals to engage meaningfully with their givens and pursue authentic lives.

Both Habermas and Fukuyama concede that therapeutic genetic engineering to address severe diseases is ethically permissible, likely recognising that outright rejection would be viewed as excessively rigid and lacking compassion. This concession highlights the difficulty in maintaining the pure essentialist position that the species‐typical human genome has inherent value and thus categorically rejects all genetic engineering. Furthermore, the therapeutic genetic engineering concession draws attention to a broader issue: the counterfactual nature of the very notion of the inherent value of the species‐typical human genome. Genetic predispositions to complex diseases are widespread across the population—research has shown that polygenic risk scores highlight the cumulative effect of common genetic variants on the risk of developing serious conditions such as cancer, cardiovascular disease, and neurodegenerative disorders [17]. These predispositions are not rare anomalies but are intrinsic to the general population's genetic makeup, with genetic risk broadly distributed and significantly influencing health outcomes across individuals [18]. The species‐typical human genome, far from being an ideal, is fraught with potential health issues. Thus, Habermas and Fukuyama's concession exposes the fragility of essentialism. Moreover, it sets an ethical precedent: if certain kinds of genetic engineering can be permissible without compromising autonomy or dignity, then the rigid essentialist assumption that all genetic engineering necessarily undermines these values becomes untenable. This reveals that the real issue is not the act of genetic engineering itself but the ethical context and purpose within which it is applied.

In contrast, existentialist philosophy offers a more nuanced and ethically robust framework. It acknowledges that genetic engineering, like any parental choice, can either constrain or enhance autonomy depending on how it is employed. Authenticity, in this view, is a dynamic, relational, and inclusive process. This perspective shifts the ethical debate from a blanket condemnation of genetic engineering to a focus on its thoughtful integration as a tool for fostering human flourishing.

By reframing the debate, existentialism moves beyond the static, essentialist assumptions of Habermas and Fukuyama to a more dynamic understanding of identity and intergenerational responsibility. Instead of seeing genetic engineering as a determinant of identity, the focus becomes creating conditions that support the child's future autonomy and ability to engage meaningfully with their genetic and environmental givens. Parents play a critical role as co‐creators of their child's initial conditions, and their responsibility lies in establishing a foundation that fosters the child's capacity for authentic engagement with their inherited traits. Thus, genetic engineering, when approached thoughtfully, is not a tool for imposing fixed identities but for empowering future generations to pursue autonomy and flourishing.

## 4 Authenticity in the Age of Genetic Engineering: Aspirations and Limits

### 4.1 The Promise of Greater Human Flourishing

The possibility of genetic engineering represents a profound leap in procreative autonomy, empowering parents to shape their children's foundational traits in alignment with their deepest values and aspirations. This revolutionary technology transcends the current limitations of parenting, which are restricted to environmental and psychosocial influences post‐conception. Instead, genetic engineering enables parents to mould the ‘clay’ of their children's potential before birth, offering a more intentional and values‐driven approach to procreation. Thoughtfully applied, genetic engineering can not only preserve but also expand a child's capacity for self‐expression and identity formation by enhancing his or her opportunities for flourishing. For instance, genetic modifications that improve cognitive abilities, physical health, or resistance to debilitating diseases can provide the child with greater freedom to explore diverse paths and realise their full potential.

Existentialist philosophers such as Heidegger and Sartre emphasise that living authentically requires certain essential capacities: the ability to make meaningful, non‐trivial choices, engage with one's existence, and take responsibility for one's actions. These capacities form the foundation of an authentic life. Importantly, existentialist philosophy not only accommodates but actively supports genetic enhancements that strengthen these capacities. By enhancing traits that enable individuals to make informed decisions and engage meaningfully with the world—such as improved cognitive function or emotional resilience—genetic engineering aligns with existentialist principles of autonomy and self‐realisation. This, in turn, promotes human flourishing, which is a fundamental ethical goal.

This alignment with human flourishing strengthens the case for existentialism as an ethically superior framework to essentialism. Where essentialism resists genetic interventions as threats to a fixed human essence, existentialism embraces the transformative potential of these technologies. By not only tolerating but affirming the value of genetic engineering as a means to enrich autonomy, self‐expression, and human flourishing, existentialism offers a dynamic and inclusive ethical approach. This highlights why existentialism is not just an alternative to essentialism but a more attractive framework for guiding the ethical use of genetic engineering.

### 4.2 Contours of Procreative Autonomy

While genetic engineering holds the promise of enhancing human capabilities, it must also be critically evaluated for its potential to impose constraints on genetically engineered individuals. Two key ethical principles provide contrasting approaches to parental decision‐making in the context of genetic interventions: the Principle of Procreative Beneficence, which aims at optimising the child's well‐being, and the Principle of Procreative Non‐Maleficence, which prioritises avoiding harm and safeguarding autonomy.

#### 4.2.1 The Principle of Procreative Beneficence

Julian Savulescu proposed the Principle of Procreative Beneficence, which argues that parents have a moral obligation to select the child most likely to have the best possible life, based on available genetic information [19]. Under the Principle of Procreative Beneficence, the use of genetic enhancements aligns with this responsibility, as it aims to improve traits that could support the child's capacity for a fulfilling life. However, the Principle of Procreative Beneficence may conflict with authenticity if optimising for specific traits limits the child's autonomy and ability to make free, self‐determined choices. For example, consider a society—contemporary Western society?—that places a high value on cooperativeness and amicability, where individuals who are non‐confrontational and highly agreeable are more likely to thrive and experience social acceptance. In this context, parents might genetically engineer their child to embody these traits, ensuring that the child's personality aligns with what would maximise its well‐being in such a society. However, this genetic programming would also constrain the child's autonomy by making it exceedingly difficult, or even impossible, for it to adopt a more assertive or challenging role.

#### 4.2.2 The Principle of Procreative Non‐Maleficence

Donrich Thaldar and Bongi Shozi propose the Principle of Procreative Non‐Maleficence, a sufficing principle that focuses on avoiding harm rather than optimising outcomes [20]. According to the Principle of Procreative Non‐Maleficence, prospective parents have a moral obligation to ensure that their procreative decisions do not result in significant harm or unduly restrict the child's future autonomy. The emphasis is on providing the child with at least the ‘ordinary chances of a desirable existence,’ which echoes Joel Feinberg's concept of the right to an open future [21]. The Principle of Procreative Non‐Maleficence aligns closely with the capacities necessary for authenticity, as it ensures that the child retains the ability to make meaningful life choices and engage with the world.

### 4.3 The Call of Responsibility: Shaping Identity Without Dictating Destiny

The ethical evaluation of these principles gains depth when viewed through the lens of Heidegger's concept of *enframing* (*Gestell*). Introduced in his essay *The Question Concerning Technology*, enframing represents a perspective that reduces beings, including humans, to mere resources, prioritising utility over authenticity [22].^7^ Unlike the themes of *Being and Time* [1], which focus on the individual's relationship with existence, enframing shifts the focus to the systemic ways in which technology and modernity shape our understanding of the world and of ourselves. Heidegger's critique does not lie in the act of shaping children to align with societal or parental intentions—after all, children are inherently influenced by such intentions—but in the danger of applying genetic engineering in ways that strip them of their inherent dignity and freedom. The concern arises when genetic interventions rigidly dictate the conditions of children's existence, reducing their lives to predefined roles and purposes, thereby undermining their ability to live authentically.^8^

The Principle of Procreative Beneficence prioritises optimising well‐being, urging parents to select genetic traits that offer children the best possible life. This principle has significant ethical value, as it highlights the potential of genetic engineering to enhance human flourishing through improvements in traits such as cognitive abilities, health, and resilience. However, Heidegger's critique of enframing warns against a purely instrumental approach to this principle. If genetic interventions predetermine children's paths and leave no room for authentic self‐definition, they risk being confined to modes of existence that prioritise societal or parental ideals over their own capacity for autonomy and authenticity. For instance, while enhancing traits like intelligence or musical talent might expand opportunities for self‐expression, programming children to exhibit extreme cooperativeness or reduced aggressiveness could confine them to specific roles or expectations, limiting their ability to redefine themselves. The issue is not enhancement itself but the extent to which it precludes children's capacity for an authentic and meaningful life.

Heidegger's insights underscore the ethical tension within the Principle of Procreative Beneficence. While the principle seeks to optimise children's well‐being, its application must be tempered by the Principle of Procreative Non‐Maleficence, which ensures that genetic enhancements safeguard autonomy. By preserving an open future, this principle allows children to navigate their own paths and engage authentically with their existence.

It is essential to consider Sartre's philosophy alongside Heidegger's critique of enframing, as it underscores the profound responsibility inherent in freedom. Sartre's concept of *bad faith*—the denial of one's freedom and responsibility—provides a crucial lens for evaluating parental decisions in the context of genetic engineering. As explored earlier, bad faith manifests not only in blindly conforming to societal norms but also in evading the transformative opportunities presented by safe and effective genetic technologies. Sartre's framework reminds us that avoidance or denial is itself a choice and one that shirks the responsibility that comes with freedom.

In the context of human genetic engineering, the metaphor of the apple from the tree of knowledge captures this responsibility vividly. Once the knowledge and technology exist, parents cannot simply ignore them or pretend the transformative possibilities they offer do not exist. Just as one cannot return a plucked apple to the tree, the advent of genetic engineering demands that parents confront the ethical implications of their freedom to act. Choosing to avoid genetic interventions due to fear of change, passive conservatism, or a desire to preserve traditional norms constitutes bad faith, as it denies the responsibility to critically and thoughtfully engage with the possibilities available.

This perspective aligns most closely with the Principle of Procreative Beneficence, which emphasises the moral obligation to select traits that optimise the child's well‐being and potential for a flourishing life. Sartre's emphasis on radical freedom challenges parents to consider how failing to engage with genetic engineering—particularly when it offers the opportunity to enhance traits that could significantly improve the child's quality of life—reflects an abdication of their ethical responsibility. Inaction in this context risks missing opportunities to optimise the child's flourishing, which undermines the ethos of beneficence rather than non‐maleficence.

Authenticity, as Sartre frames it, requires actively engaging with the conditions of existence, including those shaped by genetic interventions. Parents who thoughtfully embrace their freedom and responsibility in these decisions can ensure that their choices enhance their child's capacity for self‐realisation and flourishing. This approach tempers the Principle of Procreative Beneficence by integrating safeguards from the Principle of Procreative Non‐Maleficence, ensuring that the focus on optimisation does not compromise the child's autonomy.^9^

Through the integration of Heidegger's and Sartre's insights, an ethical framework emerges that balances the transformative potential of genetic engineering with the imperative to respect autonomy and authenticity. Heidegger cautions against viewing genetic engineering as a mere optimisation tool, while Sartre emphasises the necessity of reflective and responsible engagement with these technologies. Together, these philosophies provide a foundation for genetic interventions that are not only transformative but also ethically grounded, fostering conditions for authentic and meaningful lives.

## 5 Conclusion

This article has explored the ethical implications of genetic engineering through the lens of authenticity, advocating for an existentialist framework as a more compelling alternative to essentialism. While essentialism categorically rejects genetic engineering as inherently compromising ethical values such as dignity and authenticity, existentialism takes a more evaluative stance. It assesses interventions based on their alignment with values like autonomy and authenticity, avoiding the deterministic assumptions that essentialism imposes.

Existentialism emphasises individual freedom, self‐determination, and the dynamic process of creating meaning in life. Unlike essentialist critiques, which falter when conceding the permissibility of therapeutic genetic engineering—undermining the idea of an inherently valuable species‐typical human genome—existentialism affirms the transformative potential of genetic engineering. It views these technologies as tools to expand autonomy, self‐expression, and opportunities for flourishing, provided they are applied thoughtfully and ethically.

Drawing on the philosophies of Heidegger and Sartre, this article demonstrates how existentialism reframes authenticity as a relational and evolving process rather than adherence to a fixed essence. Heidegger's concept of enframing warns against reducing existence to mere resources, and Sartre highlights the interplay of facticity and freedom, advocating for responsible engagement in shaping one's identity. These perspectives illustrate that genetic interventions, when guided by existentialist values, can support rather than undermine authentic self‐realisation.

The metaphor of Zazu, my dachshund, illustrates that transformation—whether through natural evolution or human intervention—need not compromise authenticity. This insight underscores that a posthuman child's authenticity can similarly thrive within conditions shaped by ethical genetic interventions. To ensure ethical practice, genetic engineering must balance aspirations for human flourishing with the imperative to preserve autonomy and avoid harm. This balance is best achieved through the complementary application of Procreative Beneficence and Procreative Non‐Maleficence. Procreative Beneficence encourages thoughtful enhancements that expand opportunities for flourishing, while Procreative Non‐Maleficence ensures that such interventions do not impose deterministic constraints or compromise autonomy. Together, these principles create a framework that aligns genetic interventions with existentialist values.

By integrating these principles, existentialism offers a nuanced approach that avoids essentialism's categorical rejection of genetic engineering and the risks of uncritical optimisation. This approach ensures that genetic enhancements function as resources for self‐creation rather than constraints, preserving the child's capacity for meaningful choice and narrative identity. The existentialist framework provides the ethical foundation to guide these interventions, promoting a vision of genetic innovation that respects both autonomy and the conditions necessary for authentic living.

## Data Availability

Data sharing is not applicable to this article as no new data were created or analysed in this study.

## Endnotes

1 Iftode and his colleagues underscore how significant others co‐author an individual's narrative identity, reinforcing the relational underpinnings of autonomy. See Iftode et al. [5].

2 Iftode highlights that authenticity involves both internal coherence and external experimentation, resonating with Leuenberger's conception of autonomy as a narrative process. See Iftode et al. [7].

3 For a critique of deterministic views of authenticity, see Resnik and Vorhaus [9]. For a discussion of identity as a dynamic and extended concept, grounded in autobiographical memory and interpersonal interactions, rather than as a rigid, static essence, see Lavazza [10].

4 DeGrazia highlights that identity is shaped by diverse influences, arguing that genetic engineering, like educational or cultural choices, provides a framework for self‐expression rather than dictating it. See DeGrazia [13].

5 For a comprehensive critique of Fukuyama, see Jordaan [14].

6 Schultz‐Bergin critiques the essentialist linkage between authenticity and the unaltered state of nature, arguing that authenticity is not compromised by technological interventions but is instead constructed through evolving societal and personal frameworks. See Schultz‐Bergin [15].

7 Note that *The Question Concerning Technology*, the essay in which Heidegger introduces the concept of enframing (*Gestell*), was originally delivered as a lecture in 1949. It was later revised and published in German in 1954 as part of the collection *Vorträge und Aufsätze*. The English translation appeared in the 1977 book *The Question Concerning Technology and Other Essays*, which includes additional essays such as *The Turning* and *The Word of Nietzsche: ‘God is Dead’.*

8 Juth and Levy both examine the intersection of genetic interventions and authenticity, emphasising the importance of autonomy. Juth argues that desires shaped by genetic interventions can be authentic if they align with second‐order desires reflecting critical self‐reflection, but cautions that societal or parental expectations risk undermining autonomy. Similarly, Levy contends that genetic engineering aligns with authenticity when it fosters a child's flourishing, rather than imposing rigid expectations. See Juth [23] and Levy [24].

9 It is interesting to note that this conclusion seems to align well with the work of utilitarian ethicists such as Tännsjö and Harris. Tännsjö advocates for procreative autonomy, emphasising parental decisions that enhance a child's opportunities for flourishing while respecting ethical boundaries and avoiding deterministic constraints. See Tännsjö [25]. Similarly, Harris highlights the ethical potential of enhancement technologies to promote autonomy and flourishing but warns against interventions that impose moral ideals could erode individual self‐determination and responsibility. See Harris [26].
